# Factors influencing quality of processing in EMDR therapy

**DOI:** 10.3389/fpsyg.2024.1432886

**Published:** 2024-09-02

**Authors:** Alejandra Ramallo-Machín, Francisco J. Gómez-Salas, Francisco Burgos-Julián, M. A. Santed-Germán, Ana Isabel Gonzalez-Vazquez

**Affiliations:** ^1^Universidade da Coruña, Facultad de Ciencias, A. Coruña, Spain; ^2^Facultad de Psicología y Logopedia, Universidad de Málaga, Málaga, Spain; ^3^Facultad de Psicología, Universidad Autónoma de Madrid, Madrid, Spain; ^4^Facultad de Psicología, Universidad Nacional de Educación a Distancia (UNED), Madrid, Spain; ^5^Departamento de Psiquiatría, Complexo Hospitalario Universitario de A. Coruña, A Coruña, Spain

**Keywords:** EMDR, trauma, emotion regulation, emotional processing, symptoms-improvement

## Abstract

This study presents a preliminary analysis of a new instrument oriented at the analysis of processes in EMDR trauma therapy, the Processing Difficulties Scale (PDS). This scale includes 17 items described by experienced EMDR consultants and practitioners as indicative of problems during memory reprocessing. The proposed factorial solution based on four factors explains a total variance explained of 55% and an adequate goodness of fit, based on the proposed indices: RMSEA = 0.07; TLI = 0.91; CFI = 0.95. Table 1 shows the factorial loads for each of the items. The first factor includes 5 items (7, 8, 9, 10, 11), the second factor includes 6 items (13, 14, 25, 27, 28, 31), the third factor includes 3 items (3, 16, 22) and the fourth factor includes 3 items (19, 23, 24). Confirmatory analysis confirms the factorial solution proposed in the exploratory analysis factor and based on four factors with 17 items. The analysis of internal consistency from Cronbach’s alpha and the Omega index shows good internal consistency: Factor 1 (good processing; α = 0.92; ω = 0.94), Factor 2 (lack of generalization and/or absence of changes; α = 0.87; ω = 0.90), Factor 3 (poor emotional processing; α = 0.83; ω = 0.85) an Factor 4 (loss of dual attention; α = 0.82; ω = 0.83). In the case of the total scale, both coefficients exceeded 0.90, with an alpha of 0.92 and an Omega of 0.94. The convergent and discriminant validity criteria were estimated by calculating correlations, exploring the relationship between the factors resulting from the final result, the global severity index (GSI) of the SCL-90 and the level of improvement (NGS). These statistical analyses showed good levels of convergent and discriminant validity for all final factors. The PDS may offer a different perspective to analyze the controversy between clinicians and researchers about the need of a preparation phase in patients with complex early traumatization, dissociative symptoms and/or emotion dysregulation, and the different results in specific research around this topic. Exploring the problems in processing in a transdiagnostic way, in a preliminary analysis, we found that the number of early traumatic events measured with the ACE correlates positively with indicators of a loss of dual attention, while emotional dysregulation measured with the DERS does not predict poor processing. Finally, the dissociation measured with the DES seems to correlate positively with the indicators of a loss of dual attention during processing, not seeming to predict poor processing but did show a negative correlation with the indicators of good general processing. These results partially support the findings of some authors on the involvement of certain variables in the processing of traumatic memories, and it may be interesting to evaluate processing styles and their relationship with various indicators, to develop specific interventions in phase 2 of EMDR therapy, thus improving clinical interventions.

## 1 Introduction

EMDR therapy (Shapiro, 1996, 1998, 2018) is a trauma-oriented treatment that has been recognized as an evidence-based therapy for post-traumatic stress disorder (PTSD) (Bisson et al., 2013). The acronym EMDR stands for Eye Movement Desensitization and Reprocessing, which refers both to the processing of memories, which is the basis on which this treatment is structured, and to the use of eye movements for this purpose. Although the debate on the role of eye movements and their mechanism of action remains open (Landin-Romero et al., 2018), what seems clear is that eye movements are an active ingredient with moderate to large effect sizes (Lee and Cuijpers, 2013).

The EMDR model posits that a relevant part of psychopathology is derived from certain experiences that cannot be processed and remain dysfunctionally stored. The basis of EMDR therapy is to identify and access these experiences. Once the memory becomes active in the nervous system, the eye movement - or other bilateral stimulation (BS) methods, such as tapping or alternating auditory stimulation—are used to unlock that memory and promote its integration into other more adaptive networks (Leeds, 2013; Shapiro, 2018; Hensley, 2020).

There is a growing amount of evidence on the effectiveness of EMDR procedures in Posttraumatic Stress Disorder (PTSD) and, increasingly, in other pathologies (Bisson et al., 2007, 2013; Acarturk et al., 2016; Chiorino et al., 2020; Carletto et al., 2021; Ironson et al., 2021; Yan et al., 2021; Burr et al., 2022; Conijn et al., 2022; Hudays et al., 2022). However, there is also a relevant individual variability in-between subjects, without to date clearly identified factors differentiating between responders and non-responders to EMDR therapy. At the same time, within this area controversy has arisen in recent years about whether a preparation phase prior to memory processing is necessary, and what it should focus on De Jongh et al. (2016). While clinicians have proposed various strategies to improve emotional regulation and work with dissociation, some authors, primarily from the research field, have provided data suggesting that emotional regulation and dissociation do not negatively influence memory processing (Van Toorenburg et al., 2020; Van Der Linde et al., 2023).

We think that part of this controversy may be due to the fact that both clinicians and researchers are working with different patients and observing different elements along the therapeutic process. Subjects who agree to take part in study aimed at working on traumatic memories present a willingness to do so that many patients who attend therapy do not present, mainly in the initial stages. In addition to the fact that many studies focus on analyzing pre-post differences in symptoms but, generally, analysis of the processes in the session are usually lacking.

In this study, we will approach the issue of the individual psychotherapy response from a different perspective. At the clinical level, EMDR therapists usually describe important differences in processing styles, which have been collected on a specific descriptive scale (Processing Difficulties Scale, PDS). The effect of bilateral stimulation usually consists of promoting an associative process, decreasing disturbance, allowing the image to become more distant, blurrier, and/or enhancing thoughts related to gaining perspective or becoming aware of other aspects. When these changes are not observed, it can be an indicator of an unproductive processing. The goal of this study is to operationalize the concept of “unproductive processing.” The scores obtained on this scale reflect the difficulties that therapists encounter in phases 4–8 of EMDR memory reprocessing. This processing style scale focuses on process analysis, rather than outcome analysis. Both aspects are complementary and necessary in each psychotherapeutic orientation, with the analysis of processes in EMDR therapy having little development to date. The present study focuses on analyzing the characteristics of the Processing Difficulties Scale (PDS).

The concept of emotional processing has been described (Rachman, 1980) as the process by which an emotional disturbance generated by a stressful life event decreases until the person can reach previous functioning. However, when emotional experiences are not fully integrated or processed, this can lead to the return of fears, or the development of obsessions or intrusive thoughts, symptoms that are included in the description of PTSD (Ehlers and Clark, 2000; Brewin, 2001). For this reason, Rachman (2001) proposed the excessive avoidance or rigid and prolonged inhibition of the negative emotional experience as factors that may prevent its reintegration and resolution.

Recently, a model for psychological trauma has been developed as a transdiagnostic construct in psychopathology, in which emotional processing constitutes a transdiagnostic risk mechanism (McLaughlin et al., 2020). Likewise, Pascual-Leone and Greenberg (2007) point out that it is important to develop the role of emotional processing in different therapeutic approaches because its nuances seem to be applied differently in different therapy models. For example, Foa and Kozak (1986) describe indicators that emotional processing has occurred—related to a gradual decrease in fear and response to the feared object—by stating that, if this curve is not seen, the fact that the object simply does not activate the previous reaction could also be due to an avoidance mechanism. With EMDR, since the underlying mechanism does not appear to be habituation but reconsolidation of memories (Suzuki et al., 2004), the decrease in emotion often occurs completely within a single session. However, when the processing is unproductive this may not occur, and there may be different factors that are influencing said unproductive processing.

Hayes et al. (2007) also notes that, in order to assess its clinical significance, researchers must develop a moment-by-moment emotional processing model that spans all the treatment approaches, including what occurs during the session. Welling (2012) proposes taking into account the sequence of emotional transformation as a common principle of change in psychotherapy, pivoting from this basis on the interventions in different models of therapy, although he points out that when there is avoidance or difficulties identifying certain emotions, it is more reasonable to use exposure therapy. This analysis of processes in psychotherapy has not been deeply discussed in the EMDR therapy literature, in which hypotheses on what active bilateral stimulation activates or triggers, and why it does, are predominant.

Taking into account what was mentioned above, it could be interesting to evaluate processing styles and their relationship with various indicators, in order to develop specific interventions in phase 2 of EMDR therapy, thus improving clinical interventions. The PDS may offer a different perspective to analyze the controversy between clinicians and researchers about the need for a preparation phase in patients with complex early traumatization, dissociative symptoms, and/or emotional dysregulation.

## 2 Materials and methods

### 2.1 Design

This study is a descriptive analysis investigating different factors related with styles of processing in patients from a transdiagnostic perspective.

### 2.2 Setting

This was a multicenter study, and therefore patients were consecutively recruited between 2019 and 2023 from different EMDR therapists.

This study was approved by the Research Ethics Committee of Galicia 2017/425. Informed written consent was obtained from all participants.

### 2.3 Participants

The participants in the study consisted of 228 patients with different psychiatric diagnoses (88 subjects presented *anxiety* disorders, 42 *depressive* disorders, 37 *posttraumatic stress* disorder, 29 other *disorders related to trauma and stress factors* and 32 other diagnoses) who were under EMDR treatment for almost 6 months. Participants were assessed using the Mini-International Neuropsychiatric Interview-Plus (MINI-Plus) (Sheehan et al., 1998) clinical interview, in order to confirm the diagnosis.

Inclusion criteria were as follows: (1) diagnosis of some type of mental disorder; (2) aged between 18 and 65 years; (3) having received EMDR treatment for at least 3 months; (4) legal capacity to consent to the treatment.

Exclusion criteria were as follows: (1) a serious, unstable medical condition; (2) inability to understand and fill out the questionnaires; and (3) lack of compliance in the psychotherapy process.

### 2.4 Recruitment and measures

A group of 25 EMDR experienced therapists participated in the study. A first stage was the definition of the variables on the scale, exploring with different practitioners and consultants which characteristics they considered typical of a “good processing” or a “bad processing.” Many characteristics were included in order to analyze this concept and have some preliminary data about factors related to the quality of processing sessions.

The scale for this study was defined for the therapist retrospectively, describing how the patient usually functions when processing traumatic memories. The therapist proposed their participation in the research protocol to patients during a routine clinical visit. The research protocol and aims of the study were explained to patients who met the inclusion/exclusion criteria. They were also told that their therapeutic process would not change whether they took part in the study or not. If they agreed, they signed the consent form, and then were asked to proceed with the psychological assessment.

The following psychological self-report questionnaires were administered:

1. *Questionnaire of general characteristics*: age, sex, educational level, therapist training level and main psychiatric diagnosis.
2. The evaluation of the general symptoms presented by the patients will be carried out using the *Revised 90 Symptom Questionnaire* (*Symptom Chekclist-90-Revised, SCL-90-R;* Derogatis, 2017). A self-administered questionnaire of 90 items that are evaluated according to a 5-point Likert-type scale, from O (absence of the symptom) to 4 (presence total of the same), depending on the discomfort you have experienced in the last week. Its application requires between 10 and 20 min, and with the correction of the test you obtain 9 symptomatic scales: somatization, obsession-compulsion, interpersonal sensitivity, depression, anxiety, hostility, phobic anxiety, paranoid ideation, psychoticism; and 3 global indices: global severity index, positive and total symptomatic discomfort index of positive symptoms. It has good psychometric properties. For its evaluation and quantification, the items corresponding to each symptomatic scale are added obtaining a value for each factor. Regarding the indices, for the Severity Index Globally (GSI), the scores obtained in the nine symptom dimensions and in the additional items are added, and that number is divided by the total number of responses given; in the index of positive symptoms, responses that are different from zero are counted; and the index of positive discomfort. It is calculated by dividing the total sum of the responses given to the items by the value obtained in Total Positive Symptoms.
3. To evaluate the level of global evolution of the participants, since the beginning of EMDR therapy, the therapists, in addition to the subjective assessment, quantified this variable through the use of the Global Assessment of Functioning Scale (GAF) (American Psychiatric Association, 1994), in two moments: at the beginning of EMDR therapy and when completing the questionnaire. GAF is a descriptive scale that provides a single score to assess subjects’ level of psychological, social, and occupational functioning along a hypothetical health-illness continuum (1–100). A higher score is interpreted as a better activity level.

In order to explore the process in the therapy session, we have defined a new analytic tool: the Processing Difficulties Scale (PDS) defined, initially, by 32 that are associated with different processing styles: indicators of *poor processing* with lack of generalization (the associations during the intervention they do not generalize to other experiences, there are blockages or “loopings” during processing, difficulty in installing positive beliefs or in connecting the patient with adaptive information); *good general processing* (adequate installation of positive belief, the memory is processed completely during the session, changes are detected in the subjective meaning of the experience, etc.); *unproductive emotional processing* (avoidance or refusal to experience certain emotions, the therapist’s use of weaving does not facilitate processing) and *Indicators of a loss of dual attention* during the session.

## 3 Data analysis

Based on the minimum recommended criteria for sample size (>200) and following the rule of 10 subjects per variable, a minimum required size of 200 subjects is estimated for the exploratory factor analysis (Martínez-Arias et al., 2007). A prior descriptive and exploratory analysis procedure is carried out with the objective of checking the adequacy of the data to a multivariate normal distribution (Royston test). From the calculation of the Kendall correlation coefficient, the correlation matrix between each of the items is estimated. The Bartlett test of sphericity and the Kaiser-Meyer-Olkin test (KMO) allow us to test the hypothesis of independence of the scale elements and, therefore, verify the suitability for factorization.

The determination of the number of factors to extract is based on the Kaiser criterion for those factors that show eigenvalues above 1, complemented by the scree plot. Exploratory factor analysis (EFA) uses a robust estimation method using the minimum residual (minres) criterion and a Promax factor rotation method. To reduce the scale, the following criteria were established: (a) a factor loading greater than 0.50; (b) when saturation occurs in two or more factors, it will be retained in the factor with the highest saturation, as long as the values of the factor loadings in several factors do not exceed 0.50, in which case it will be eliminated; (c) elimination of factors with less than three items; (d) elimination of factors with less than 5% of explained variance.

The analysis of the goodness of fit of the model to the data was carried out using the comparative fit index (CFI), the Tucker-Lewis index (TLI) and the root mean square error of approximation (RMSEA) as estimators. For the CFI and TLI indices, values above 0.90 are recommended, while for the RMSEA, values below 0.60 are recommended (Hu and Bentler, 1999). The analysis of the internal consistency of the questionnaire is carried out using Cronbach’s alpha coefficient and the Omega index, with values greater than 0.70 indicating adequate reliability. The convergent and discriminant validity criteria were estimated by calculating correlations, exploring the relationship between the factors resulting from the final result, the global severity index (GSI) of the SCL-90 and the level of improvement (NGS), which is processed using the following calculation: Final severity—Initial severity/number of sessions. On the other hand, the potential relationship between the variables sex, age, educational level and training of the therapist is evaluated by estimating a multiple regression model. All calculations were performed using R software (R Core Team, 2014).

## 4 Results

### 4.1 Preliminary descriptive and exploratory analysis

The sample is made up of a total of 228 people, with an age range between 18 and 66 years (M = 37.65; SD = 10.57), of which, 190 are women (83.33%) and 38 men (16.67%), while 5 have primary studies (2.19%), 40 secondary studies (17.54%) and 183 higher studies (80.26%). The exploratory analysis showed a significant value for Royston’s multivariate normality test (*R* = 1058.78; *p* < 0.05) so the use of robust methods in the factorization process is recommended.

The KMO test shows values greater than 0.80 (KMO = 0.93), while Bartlett’s sphericity test (χ^2^ = 5501.74, *p* < 0.01), shows statistically significant results, which ensures the absence of independence between the items and the adequacy of the correlation matrix for subsequent factorization.

### 4.2 Exploratory factor analysis

**Figure 1:**
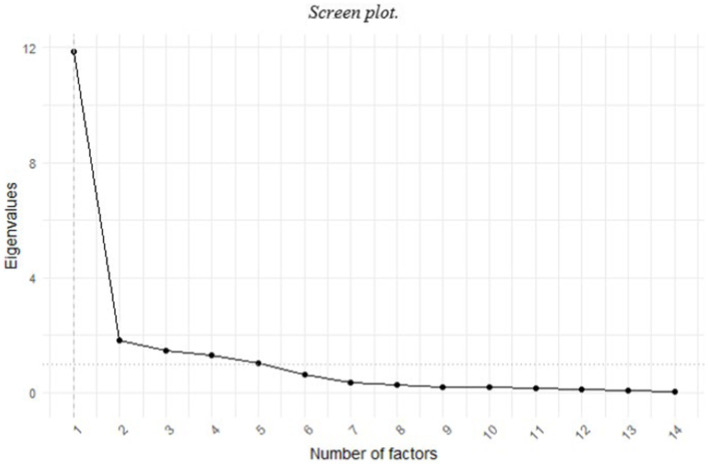
Screen plot.

**Figure 2:**
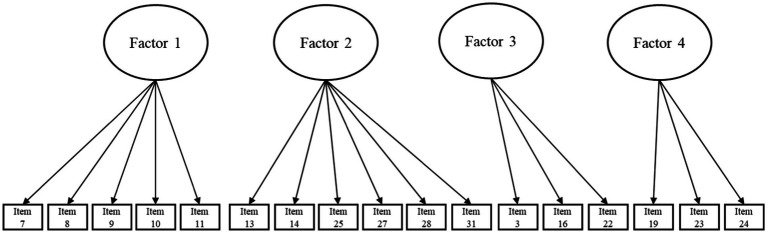
Graphical representation of the final factor model.

The screen plot allowed us to observe five factors with eigenvalues above 1 (see Figure 1). In the process of retaining items from the scale, those that had factorial weights lower than 0.50 were first eliminated. Additionally, the composition of the emerging factors was reviewed, eliminating any factor that contained less than three items to guarantee the stability and interpretability of each factor. Following this criterion, an entire factor containing only two items was excluded. Consequently, the factor structure was adjusted to four main factors. Therefore, and based on the item retention criteria and refinement of the factor structure, the initial scale composed of 32 items was converted to a final solution of 17 grouped into four factors (see Figure 2), with a total variance explained of 55% and an adequate goodness of fit, based on the proposed indices: RMSEA = 0.07; TLI = 0.91; CFI = 0.95 (see Table 1). As, a description of the items of the resulting scale, their correspondence with the original scale and the factor to which each item belongs is provided (see Table 2).

**Table 1:** Factor loadings for the final solution based on four factors.

Items	Factor			
	1	2	3	4
7	0.94			
8	0.99			
9	0.65			
10	0.53			
11	0.66			
13		0.55		
14		0.58		
25		0.66		
27		0.71		
28		0.62		
31		0.87		
3			0.53	
16			0.88	
22			0.69	
19				0.57
23				0.76
24				0.78

Factor weights in absolute values.

**Table 2:** Correspondence between PDS scale, initial questionnaire and factor number.

Processing Difficulties Scale (PDS) ítems (17)	Initial questionnaire ítems (32)	Factor
1. Different emotions appear, which the patient allows to arise, flow and evolve	3	3
2. Positive cognition installs easily	8	1
3. The image remains unchanged	25	2
4. He immerses himself completely in the memory, he seems not to realize that he is in the office and in the present	23	4
5. Many rounds are required in relation to other patients to be able to establish the positive belief	28	2
6. The VOC upon reaching the installation phase of the positive belief is 5 or more	7	1
7. The therapist must carry out interventions to maintain dual attention	24	4
8. In phase 6, the body check presents a mild disturbance that can be easily processed, a total absence of disturbance, or a clearly positive body sensation	9	1
9. Marked depersonalization/derealization or intrusions of dissociative parts appear	19	4
10. Associative chains are extremely long and take time to connect with adaptive information, or do not connect with this information	13	2
11. Has difficulty connecting emotionally with the memory or disconnects during processing	22	3
12.The patient states that his belief will never change, or seems to cling to it	27	2
13. In phase 8 it is verified that the memory is completely processed	11	1
14. The subjective meaning of the experience remains similar to the beginning of the session	31	2
15.There are certain emotions that the patient avoids or rejects, or specific emotions that would make sense to appear due to the characteristics of the memory do not appear	16	3
16. The associations do not generalize to other experiences, emotions or sensations, they remain very directly connected to the target event	14	2
17. When processing a memory, the subjective meaning of the experience changes	10	1

The clinician evaluates the work with EMDR, following the standard protocol, answering the ítems of the PDS questionnaire according to the scale: 0 = Never; 1 = Almost Never, 2 = Sometimes; 3 = Quite a few times and 4 = Always.

### 4.3 Reliability analysis

The internal consistency analysis was carried out using Cronbach’s alpha coefficient and the Omega index; both indicators revealed good internal consistency with values greater than 0.70 in all cases. Specifically, Factor 1 showed a Cronbach’s alpha of 0.92 and an Omega index of 0.94. Factor 2 recorded values of 0.87 and 0.90 for alpha and Omega, respectively. For Factor 3, values of 0.83 for alpha and 0.85 for Omega were obtained. Factor 4 presented an alpha of 0.82 and an Omega of 0.83. In the case of the total scale, both coefficients exceeded 0.90, with an alpha of 0.92 and an Omega of 0.94.

### 4.4 Convergent and discriminant validity

In the study of correlations to evaluate the convergent and discriminant validity of the factors with respect to the GSI and the NGS, significant patterns of association were observed. Factor 1 shows a significant negative correlation with the GSI (*r* = −0.34, *p* < 0.01, 95% CI [−0.52, −0.13]), suggesting discriminant validity between these two constructs. In contrast, Factor 1 correlates positively with the NGS (*r* = 0.32, *p* = 0.01, 95% CI [0.11, 0.50]), showing evidence of convergent validity. Regarding Factor 2, a positive correlation is observed with the GSI (*r* = 0.32, p = 0.01, 95% CI [0.11, 0.50]) and a negative correlation with the NGS (*r* = −0.36, *p* < 0.01, 95% CI [−0.54, −0.16]). These correlations indicate evidence of convergent validity with the GSI and discriminant validity with the NGS (see Table 3).

**Table 3:** Means, standard deviations, and correlations with confidence intervals.

Variable	*M*	*SD*	1	2	3	4	5
1. Factor 1	11.55	6.45					
2. Factor 2	9.20	5.45	−0.64**				
			[−0.75, −0.49]				
3. Factor 3	5.63	1.69	−0.45**	0.55**			
			[−0.60, −0.25]	[0.37, 0.68]			
4. Factor 4	3.50	3.06	−0.48**	0.32**	0.31**		
			[−0.63, −0.29]	[0.11, 0.50]	[0.10, 0.49]		
5. GSI	1.48	0.78	−0.34**	0.32**	0.34**	0.33**	
			[−0.52, −0.13]	[0.11, 0.50]	[0.13, 0.52]	[0.12, 0.51]	
6. NGS	1.58	2.77	0.32**	−0.36**	−0.41**	−0.33**	−0.18
			[0.11, 0.50]	[−0.54, −0.16]	[−0.57, −0.21]	[−0.51, −0.12]	[−0.38, 0.04]

M and SD are used to represent mean and standard deviation, respectively. Values in square brackets indicate the 95% confidence interval for each correlation. The confidence interval is a plausible range of population correlations that could have caused the sample correlation (Cumming, 2014). *p < 0.05. **p < 0.01. GSI, Severity Index Globally; NGS, The level of improvement.

Factor 3 also shows significant positive correlations with both the GSI (*r* = 0.34, *p* < 0.01, 95% CI [0.13, 0.52]) and the NGS (*r* = −0.41, *p* < 0.01, 95% CI [−0.57, −0.21]), reflecting convergent validity with the GSI and discriminant validity with the NGS. Finally, Factor 4 presents positive correlations with the GSI (*r* = 0.33, *p* < 0.01, 95% CI [0.12, 0.51]) and negative correlations with the NGS (*r* = −0.33, *p* < 0.01, 95% CI [−0.51, −0.12]), once again providing evidence of convergent and discriminant validity (see Table 3).

### 4.5 Instruments scores

Table 4 shows the results of the multiple regression analysis for each factor resulting from the factor solution. Each of the factors resulting from the proposed solution is introduced into the model as a criterion variable. The *p* value is corrected using the Bonferroni correction. Sex, age, educational level and level of training of the therapists are introduced as predictor variables. While, as criterion variable, the respective factors resulting from the EFA are introduced.

**Table 4:** Regression results using the factors as the criterion.

Predictor	*b*	*b* 95% CI [LL, UL]	*Beta*	*Beta* 95% CI [LL, UL]	*sr^2^*	*sr^2^* 95% CI [LL, UL]	*r*	Fit
Factor 1								
Sex	−0.87	[−4.51, 2.76]	−0.05	[−0.26, 0.16]	0.00	[−0.02, 0.02]	−0.07	
Age	0.04	[−0.09, 0.17]	0.07	[−0.14, 0.28]	0.00	[−0.02, 0.03]	0.07	
Education level	−1.75	[−4.47, 0.97]	−0.14	[−0.35, 0.08]	0.02	[−0.03, 0.07]	−0.07	
Training level	−3.83**	[−5.39, −2.27]	−0.52	[−0.73, −0.31]	0.26	[0.09, 0.44]	−0.50**	
								*R^2^* = 0.278**
								95% CI [0.08, 0.40]
Factor 2								
Sex	−1.03	[−4.53, 2.47]	−0.07	[−0.31, 0.17]	0.00	[−0.03, 0.04]	−0.08	
Age	0.05	[−0.07, 0.18]	0.10	[−0.13, 0.34]	0.01	[−0.04, 0.06]	0.11	
Education level	−0.18	[−2.80, 2.45]	−0.02	[−0.26, 0.22]	0.00	[−0.01, 0.01]	−0.05	
Training level	1.45	[−0.05, 2.95]	0.23	[−0.01, 0.47]	0.05	[−0.05, 0.15]	0.23	
								*R^2^* = 0.071
								95% CI [0.00,0.16]
Factor 3								
Sex	−0.02	[−1.02, 0.99]	−0.00	[−0.23, 0.22]	0.00	[−0.00, 0.00]	0.01	
Age	−0.01	[−0.05, 0.02]	−0.09	[−0.31, 0.14]	0.01	[−0.03, 0.04]	−0.08	
Education level	−0.06	[−0.82, 0.69]	−0.02	[−0.24, 0.21]	0.00	[−0.01, 0.01]	−0.07	
Training level	0.78**	[0.35, 1.22]	0.41	[0.18, 0.63]	0.16	[0.01, 0.32]	0.41**	
								*R^2^* = 0.176*
								95% CI [0.01, 0.30]
Factor 4								
Sex	0.76	[−1.16, 2.69]	0.09	[−0.14, 0.32]	0.01	[−0.03, 0.05]	0.10	
Age	−0.02	[−0.09, 0.05]	−0.07	[−0.30, 0.16]	0.00	[−0.03, 0.04]	−0.07	
Education level	−0.43	[−1.87, 1.02]	−0.07	[−0.30, 0.16]	0.00	[−0.03, 0.03]	−0.10	
Training level	1.10**	[0.28, 1.93]	0.31	[0.08, 0.54]	0.09	[−0.03, 0.22]	0.32**	
								*R^2^* = 0.121
								95% CI [0.00, 0.23]

A significant b-weight indicates the beta-weight and semi-partial correlation are also significant. b represents unstandardized regression weights. Beta indicates the standardized regression weights. *sr*^2^ represents the semi-partial correlation squared. r represents the zero-order correlation. LL and UL indicate the lower and upper limits of a confidence interval, respectively. Intercept values are ignored. *p < 0.05. **p < 0.01.

For Factor 1, a significant relationship was found between the level of training in EMDR therapy (*b* = −3.83, *t* = −4.91, se = 0.78, *p* < 0.01, CI [−5.39, −2.27]). This same variable has shown a significant relationship with Factor 3 (*b* = 0.78, *t* = 3.63, se = 0.22, *p* < 0.01, CI [0.35, 1.22]) and with Factor 4 (*b* = 1.10, *t* = 2.66, se = 0.41, *p* = 0.01, CI [0.28, 1.93]). While for Factor 2, no statistically significant effects have been found (see Table 4).

## 5 Discussion

There is intense debate in the scientific community about the need for a preparation phase previous to the work with traumatic memories, especially in patients with complex trauma, usually derived from early and severe interpersonal adverse situations. Some authors stress the importance of this preparation phase (Cloitre et al., 2012a) while others argue that the evidence does not support this claim, and that traumatic memories can be addressed from the very beginning of the therapeutic process (De Jongh et al., 2016; Van Vliet et al., 2021). The same debate has been established around working with dissociative disorders (Cloitre et al., 2012b; Bae et al., 2016; Zoet et al., 2018; Van Der Linde et al., 2023), and with regard to emotional dysregulation (Van Toorenburg et al., 2020). Some results support the notion that the severity of emotion regulation difficulties is not associated with worse trauma-focused treatment outcomes for PTSD. Further, emotion regulation difficulties improved after trauma-focused treatment, even for individuals who had been exposed to early childhood sexual trauma and individuals with a dissociative subtype (Van Toorenburg et al., 2020).

This debate has become more polarized among researchers and clinicians, the latter claiming that research studies do not reflect the reality of consultations with their patients and the first ones considering the clinicians opinions as subjective and against the empirical data. In general, clinicians are more favorable to progressive approaches to the treatment of patients with early traumatization, emotional dysregulation and dissociation. These discrepancies may be due on the one hand to the fact that the scientific studies usually work on samples with well-established diagnoses, while in consultation there are patients with less clear diagnoses and high comorbidity. Another element that can explain these different perspectives is the one we analyze in this article: it is possible that clinicians are more aware of the difficulties that arise in processing than the global evolution of their patients.

Process analysis can be an interesting avenue within EMDR therapy research, which can help establish specific interventions in the preparation phase with the aim of improving the processing of traumatic memories, in addition to better understanding what factors may be involved related to the difficulties that may appear during reprocessing. This perspective may fit with Hayes et al. (2007), who point out the need to carry out an analysis of what happens moment by moment in the session. That is why we believe that the scale we are trying to study can shed light in this area.

In the present study, exploratory factor analysis (EFA) of the Processing Difficulties Scale (PDS) yielded four factors that reflect different difficulties that can occur during the processing of traumatic memories in EMDR therapy: (Factor 1) indicators of good processing; (Factor 2) indicators of a lack of generalization and/or absence of changes during processing; (Factor 3) indicators of poor emotional processing; (Factor 4) indicators of a loss of dual attention.

We consider it important, in turn, to include patients with diverse diagnoses in our study patients with diverse diagnoses, exploring the problems in processing in a transdiagnostic way. In a preliminary analysis of the present scale, which will be presented in a poster at the European EMDR Congress (Ramallo-Machín et al., 2024), we carried out a correlational analysis between the four factors of the present study and the following scales: Difficulties in Emotion Regulation Scale (DERS) (Gratz and Roemer, 2004); Dissociative Experiences Scale (DES) (Bernstein and Putnam, 1986); Adverse Childhood Experiences International Questionnaire (ACE-IQ) (World Health Organization 2018).

From the results obtained in the correlational analysis between the four factors and the scales used, it seems that emotional dysregulation measured with the DERS does not predict poor processing. On the other hand, the number of early traumatic events measured with the ACE is only related to the indicators of a loss of dual attention (Factor 4), in the sense that the greater the number of early traumatic events, higher the probability of a loss of dual attention and dissociation during processing. Finally, the dissociation measured with the DES is related to the general indicators of good processing (Factor 1), in the sense that the lower the levels of dissociation, the greater the probability of good processing during the session. However, it does not seem to be related to indicators of a lack of change or generalization (Factor 2) during processing. Likewise, it is related to the indicators of a loss of dual attention (Factor 4), in the sense that the higher the level of dissociation, the greater the probability of a loss of dual attention during processing. These results partially support the findings of the aforementioned authors, and may provide a new perspective from which to analyze this controversy.

It could be interesting to evaluate processing styles and their relationship with various indicators, in order to develop specific interventions in phase 2 for EMDR therapy, as well as during the processing of traumatic memories, improving clinical interventions. The PDS may offer a different perspective to analyze the controversy between clinicians and researchers about the need for a preparation phase in patients with complex early traumatization, dissociative symptoms, and/or emotional dysregulation.

## Data availability statement

The original contributions presented in the study are included in the article/supplementary material, further inquiries can be directed to the corresponding author.

## Ethics statement

The studies involving humans were approved by Opinion of the Research Ethics Committee of A Coruña Ferrol Department of Health, Xunta de Galicia. The studies were conducted in accordance with the local legislation and institutional requirements. The participants provided their written informed consent to participate in this study.

## Author contributions

AR-M: Conceptualization, Data curation, Investigation, Resources, Writing – original draft, Writing – review & editing. FG-S: Conceptualization, Data curation, Investigation, Resources, Writing – original draft, Writing – review & editing. FB-J: Conceptualization, Formal analysis, Methodology, Resources, Software, Validation, Writing – original draft, Writing – review & editing. MS-G: Investigation, Resources, Writing – original draft, Writing – review & editing. AG-V: Conceptualization, Formal analysis, Investigation, Project administration, Resources, Supervision, Validation, Writing – original draft, Writing – review & editing.

## References

[ref1] Acarturk C. Konuk E. Cetinkaya M. Senay I. Sijbrandij M. Gulen B.. (2016). The efficacy of eye movement desensitization and reprocessing for post-traumatic stress disorder and depression among Syrian refugees: results of a randomized controlled trial. Psychol. Med. 46, 2583–2593. doi: 10.1017/S0033291716001070, PMID: 27353367

[ref2] American Psychiatric Association (1994). DSM-IV: diagnostic and statistical manual of mental disorders. Washington: APA.

[ref3] Bae H. Kim D. Park Y. C. (2016). Dissociation predicts treatment response in eye-movement desensitization and reprocessing for posttraumatic stress disorder. J. Trauma Dissociation 17, 112–130. doi: 10.1080/15299732.2015.1037039, PMID: 26156867

[ref4] Bernstein E. Putnam F. W. (1986). Development, reliability and validity of dissociation scale. J. Nerv. Ment. Dis. 174, 727–735. doi: 10.1097/00005053-198612000-00004 3783140

[ref5] Bisson J. I. Ehlers A. Matthews R. Pilling S. Richards D. Turner S. (2007). Psychological treatments for chronic post-traumatic stress disorder: systematic review and meta-analysis. Br. J. Psychiatry 190, 97–104. doi: 10.1192/bjp.bp.106.021402 17267924

[ref6] Bisson J. I. Roberts N. P. Andrew M. Cooper R. Lewis C. (2013). Psychological therapies for chronic post-traumatic stress disorder (PTSD) in adults. Cochrane Database Syst. Rev. 2013:CD003388. doi: 10.1002/14651858.CD003388.pub4, PMID: 24338345 PMC6991463

[ref7] Brewin C. R. (2001). Memory processes in post-traumatic stress disorder. Int. Rev. Psychiatry 13, 159–163. doi: 10.1080/09540260120074019

[ref8] Burr C. Schnackenberg J. K. Weidner F. (2022). Talk-based approaches to support people who are distressed by their experience of hearing voices: a scoping review. Front. Psych. 13:983999. doi: 10.3389/fpsyt.2022.983999, PMID: 36299547 PMC9589913

[ref9] Carletto S. Malandrone F. Berchialla P. Oliva F. Colombi N. Hase M.. (2021). Eye movement desensitization and reprocessing for depression: a systematic review and meta-analysis. Eur. J. Psychotraumatol. 12:1894736. doi: 10.1080/20008198.2021.1894736, PMID: 33889310 PMC8043524

[ref10] Chiorino V. Cattaneo M. C. Macchi E. A. Salerno R. Roveraro S. Bertolucci G. G.. (2020). The EMDR recent birth trauma protocol: a pilot randomised clinical trial after traumatic childbirth. Psychol. Health 35, 795–810. doi: 10.1080/08870446.2019.1699088, PMID: 31805778

[ref11] Cloitre M. Courtois C. A. Ford J. D. Green B. L. Alexander P. Briere J.. (2012a). he ISTSS expert consensus treatment guidelines for complex PTSD in adults. Available at: https://www.istss.org/ISTSS_Main/media/Documents/ISTSS-Expert-Concesnsus-Guidelines-for-Complex-PTSD-Updated-060315.pdf.

[ref12] Cloitre M. Petkova E. Wang J. Lu F. (2012b). An examination of the influence of a sequential treatment on the course and impact of dissociation among women with PTSD related to childhood abuse. Depress Anxiety 29, 709–717. doi: 10.1002/da.21920, PMID: 22550033

[ref13] Conijn T. De Roos C. Vreugdenhil H. J. I. Van Dijk-Lokkart E. M. Wijburg F. A. Haverman L. (2022). Effectiveness of time-limited eye movement desensitization reprocessing therapy for parents of children with a rare life-limiting illness: a randomized clinical trial. Orphanet J. Rare Dis. 17:328. doi: 10.1186/s13023-022-02500-9, PMID: 36056362 PMC9437394

[ref001] Cumming G. (2014). The new statistics: why and how. Psychol. Sci. 25, 7–29. 24220629 10.1177/0956797613504966

[ref14] De Jongh A. D. Resick P. A. Zoellner L. A. Van Minnen A. Lee C. W. Monson C. M.. (2016). Critical analysis of the current treatment guidelines for complex PTSD in adults. Depress Anxiety 33, 359–369. doi: 10.1002/da.22469 26840244

[ref15] Derogatis L. R. (2017). Symptom checklist-90-revised, brief symptom inventory, and BSI-18. in Handbook of psychological assessment in primary care settings, ed. Maruish M. E.. (2nd ed.), Routledge, 599–629. doi: 10.4324/9781315658407

[ref16] Ehlers A. Clark D. M. (2000). A cognitive model of posttraumatic stress disorder. Behav. Res. Ther. 38, 319–345. doi: 10.1016/S0005-7967(99)00123-0 10761279

[ref17] Foa E. B. Kozak M. J. (1986). Emotional processing of fear: exposure to corrective information. Psychol. Bull. 99, 20–35. doi: 10.1037/0033-2909.99.1.20, PMID: 2871574

[ref19] Gratz K. L. Roemer L. (2004). Multidimensional assessment of emotion regulation and dysregulation: development, factor structure, and initial validation of the difficulties in emotion regulation scale. J. Psychopathol. Behav. Assess. 26, 41–54. doi: 10.1023/B:JOBA.0000007455.08539.94

[ref20] Hayes A. M. Laurenceau J. P. Feldman G. Strauss J. L. Cardaciotto L. (2007). Change is not always linear: the study of nonlinear and discontinuous patterns of change in psychotherapy. Clin. Psychol. Rev. 27, 715–723. doi: 10.1016/j.cpr.2007.01.008, PMID: 17316941 PMC3163164

[ref21] Hensley B. J. (2020). An EMDR therapy primer: from practicum to practice (3rd ed.). Springer Publishing Company.

[ref22] Hu L. T. Bentler P. M. (1999). Cutoff criteria for fit indexes in covariance structure analysis: conventional criteria versus new alternatives. Struct. Equ. Model. 6, 1–55. doi: 10.1080/10705519909540118

[ref23] Hudays A. Gallagher R. Hazazi A. Arishi A. Bahari G. (2022). Eye movement desensitization and reprocessing versus cognitive behavior therapy for treating post-traumatic stress disorder: a systematic review and meta-analysis. Int. J. Environ. Res. Public Health 19:16836. doi: 10.3390/ijerph192416836, PMID: 36554717 PMC9778888

[ref24] Ironson G. Hylton E. Gonzalez B. Small B. Freund B. Gerstein M.. (2021). Effectiveness of three brief treatments for recent traumatic events in a low-SES community setting. Psychol. Trauma Theory Res. Pract. Policy 13, 123–132. doi: 10.1037/tra0000594 32496104

[ref25] Landin-Romero R. Moreno-Alcazar A. Pagani M. Amann B. L. (2018). How does eye movement desensitization and reprocessing therapy work? A systematic review on suggested mechanisms of action. Front. Psychol. 9:286360. doi: 10.3389/fpsyg.2018.01395, PMID: 30166975 PMC6106867

[ref26] Lee C. W. Cuijpers P. (2013). A meta-analysis of the contribution of eye movements in processing emotional memories. J. Behav. Ther. Exp. Psychiatry 44, 231–239. doi: 10.1016/j.jbtep.2012.11.001, PMID: 23266601

[ref27] Leeds A. M. (2013). Guía de protocolos estándar de EMDR para terapeutas, supervisores y consultores (2ªed.). Blioteca de psicología Desclée de Brouwer.

[ref28] Martínez-Arias M. R. Hernández Lloreda M. J. Hernández Lloreda M. V. (2007). Psicometría: Alianza Editorial.

[ref29] McLaughlin K. A. Colich N. L. Rodman A. M. Weissman D. G. (2020). Mechanisms linking childhood trauma exposure and psychopathology: a transdiagnostic model of risk and resilience. BMC Med. 18, 1–11. doi: 10.1186/s12916-020-01561-6, PMID: 32238167 PMC7110745

[ref30] Pascual-Leone A. Greenberg L. S. (2007). Emotional processing in experiential therapy: why “the only way out is through”. J. Consult. Clin. Psychol. 75, 875–887. doi: 10.1037/0022-006X.75.6.875 18085905

[ref31] R Core Team (2014). R: a language and environment for statistical computing: MSOR connections. 1.

[ref32] Rachman S. (1980). Emotional processing. Behav. Res. Ther. 18, 51–60. doi: 10.1016/0005-7967(80)90069-8 7369988

[ref33] Rachman S. (2001). Emotional processing, with special reference to post-traumatic stress disorder. Int. Rev. Psychiatry 13, 164–171. doi: 10.1080/09540260120074028

[ref34] Ramallo-Machín A. Gómez-Salas F. Burgos-Julián F. Rial-Rivas A. Santed-Germán M. A. González-Vázquez A. (2024). “The Processing Difficulties Scale (PDS)” in EMDR trauma therapy: a preliminary analysis [poster]. The EMDR Europe workshop conference (Dublin, Ireland). Available at: https://emdr24.com/

[ref35] Shapiro F. (1996). Eye movement desensitization and reprocessing (EMDR): evaluation of controlled PTSD research. J. Behav. Ther. Exp. Psychiatry 27, 209–218. doi: 10.1016/S0005-7916(96)00029-8 8959422

[ref36] Shapiro F. (1998). Eye movement desensitization and reprocessing (EMDR): accelerated information processing and affect-driven constructions: Crisis Intervention & Time-Limited Treatment. 4, 145–157.

[ref37] Shapiro F. (2018). Eye movement desensitization and reprocessing (EMDR) therapy: basic principles, protocols, and procedures (3rd ed.). The Guilford Press.

[ref002] Sheehan D. V. Lecrubier Y. Sheehan K. H. Amorim P. Janavs J. Weiller E. (1998). The mini-international neuropsychiatric interview (MINI): the development and validation of a structured diagnostic psychiatric interview for DSM-IV and ICD-10. J. Clin. Psychiatr. 59, 22–33. 9881538

[ref38] Suzuki A. Josselyn S. A. Frankland P. W. Masushige S. Silva A. J. Kida S. (2004). Memory reconsolidation and extinction have distinct temporal and biochemical signatures. J. Neurosci. 24, 4787–4795. doi: 10.1523/JNEUROSCI.5491-03.2004, PMID: 15152039 PMC6729467

[ref39] Van Der Linde R. P. Huntjens R. J. Bachrach N. Rijkeboer M. M. de Jongh A. van Minnen A. (2023). The role of dissociation-related beliefs about memory in trauma-focused treatment. Eur. J. Psychotraumatol. 14:2265182. doi: 10.1080/20008066.2023.2265182, PMID: 37846662 PMC10583636

[ref40] Van Toorenburg M. M. Sanches S. A. Linders B. Rozendaal L. Voorendonk E. M. Van Minnen A.. (2020). Do emotion regulation difficulties affect outcome of intensive trauma-focused treatment of patients with severe PTSD? Eur. J. Psychotraumatol. 11:1724417. doi: 10.1080/20008198.2020.1724417, PMID: 32166007 PMC7054933

[ref41] Van Vliet N. I. Huntjens R. J. C. Van Dijk M. K. Bachrach N. Meewisse M. L. De Jongh A. (2021). Phase-based treatment versus immediate trauma-focused treatment for post-traumatic stress disorder due to childhood abuse: randomised clinical trial. BJPsych Open 7, 1–7. doi: 10.1192/bjo.2021.1057

[ref42] Welling H. (2012). Transformative emotional sequence: towards a common principle of change. J. Psychother. Integr. 22, 109–136. doi: 10.1037/a0027786

[ref43] World Health Organization (2018). “Adverse childhood experiences international questionnaire” in Adverse childhood experiences international questionnaire (ACE-IQ), 245–258.

[ref44] Yan S. Shan Y. Zhong S. Miao H. Luo Y. Ran H.. (2021). The effectiveness of eye movement desensitization and reprocessing toward adults with major depressive disorder: a meta-analysis of randomized controlled trials. Front. Psych. 12:700458. doi: 10.3389/fpsyt.2021.700458, PMID: 34421681 PMC8377362

[ref45] Zoet H. A. Wagenmans A. Van Minnen A. De Jongh A. (2018). Presence of the dissociative subtype of PTSD does not moderate the outcome of intensive trauma-focused treatment for PTSD. Eur. J. Psychotraumatol. 9:1468707. doi: 10.1080/20008198.2018.1468707, PMID: 29805779 PMC5965028

